# *Cymbuliaparvidentata* Pelseneer, 1888 (Mollusca, Cymbuliidae) in the Ligurian Sea: further evidence of Atlantic species incursions in the Mediterranean area

**DOI:** 10.3897/BDJ.11.e99108

**Published:** 2023-02-21

**Authors:** Stefano Schiaparelli, Maria Chiara Alvaro, Alice Guzzi, Marco Grillo

**Affiliations:** 1 Department of Earth, Environmental and Life Sciences (DISTAV), University of Genoa, Corso Europa 26, 16132, Genoa, Italy Department of Earth, Environmental and Life Sciences (DISTAV), University of Genoa, Corso Europa 26, 16132 Genoa Italy; 2 Department of Physical Sciences, Earth and Environment (DSFTA), University of Siena, Strada Laterina 8, 53100, Siena, Italy Department of Physical Sciences, Earth and Environment (DSFTA), University of Siena, Strada Laterina 8, 53100 Siena Italy

**Keywords:** Cymbuliidae, Pteropoda, Ligurian Sea, stranding

## Abstract

We report the first record of a stranded specimen of *Cymbuliaparvidentata*, a pteropod species of Atlantic origin, in the Ligurian Sea. On 27 February 2022, six *C.peronii* and one *C.parvidentata* were collected on Borgio-Verezzi Beach (Savona, Italy - 44.16° N, 8.304633° W). Specimens were examined morphologically and biometrically. Measurements (length, width, height and wet weight) separated the two taxa, *C.peronii* being larger than *C.parvidentata*. The finding of *C.parvidentata*, which has only occasionally been reported in southern Italy, is remarkable and may be due to ascending Atlantic water (AW) pulses that reach the Ligurian Sea. This finding adds to the previous knowledge of other pelagic species of Atlantic origin that were found in the Ligurian Sea, suggesting the possibility of major on-going changes and a general “Atlantification”. In order to determine the frequency of such events, it will be highly desirable to design specific citizen-science campaigns.

## Introduction

The Mediterranean Sea is facing rapid changes due to increasing seawater temperatures, more frequent summer heatwaves, the arrival of alien species and increased human pressure (Fraschetti et al. 2011) which, altogether, are determining a general ecosystem shift (Bianchi et al. 2019). Despite changes in benthic ecosystems may take a longer time to occur and be documented, pelagic ecosystems are known to respond to environmental changes rather quickly (Edwards 2009), representing an ideal litmus paper of ongoing changes (e.g. Brodeur et al. (2019), Frederiksen et al. (2013)). However, monitoring at sea requires a constant effort and some sporadic events may go completely unnoticed even when rigorous sampling designs and dense temporal grid are adopted. In this context, a great help is given by the records of pelagic species strands that may bring to light changes even when these occur in off-shore water masses, notoriously more difficult to sample. In the Mediterranean area, for example, several records of stranded pelagic Atlantic species have been recently published, suggesting the existence of an ongoing Atlantification process. Atlantic pelagic species, entering through the Gibraltar Strait, follow the general trend of the currents in the north-western Mediterranean Sea (Astraldi and Gasparini 1994), where ascending Atlantic waters (AW) lap the western coast of Sardinia and enter into the Ligurian Sea. Amongst the possible examples, a notable one is that if the buoy barnacle *Dosimafascicularis* (Ellis and Solander, 1786), whose records of stranded specimens are increasingly observed (Mifsud 2005, Betti et al. 2017, Mokrane et al. 2019, Cavallaro et al. 2020). This species, typically associated to oceanic temperate waters around the world (Weisbord 1979), is a marker of Atlantic water pulses into the Mediterranean area (Mondello and Rindone 1994). Here, we report a new record of an Atlantic holoplanktonic pteropod mollusc typical of temperate waters, *Cymbuliaparvidentata* Pelseneer, 1888, that stranded in the Ligurian Sea together with the common congeneric *C.peronii* Blainville, 1818. Cymbuliidae Gray, 1840 is a family comprising the genera *Cymbulia* Péron & Lesueur, 1810, *Corolla* Dall, 1871 and *Gleba* Forsskål, 1776, where the mollusc calcareous shell is replaced by a false shell called pseudoconch (Fiasson 1970) that maintains floating capability even after the mollusc death and can be occasionally found stranded on the shore. Since Cymbuliidae represents useful bioindicators of water masses due to their sensitivity to changes in temperature, salinity and currents speed (Chen and Bé 1964), we discuss this finding in the light of a possible biogeographical interpretation of the occurrence. We also compared the two species found from a biometric point of view to provide more tools to distinguish the two taxa in the light of possible monitoring efforts in future.

## Materials and Methods

**Figure 1.**
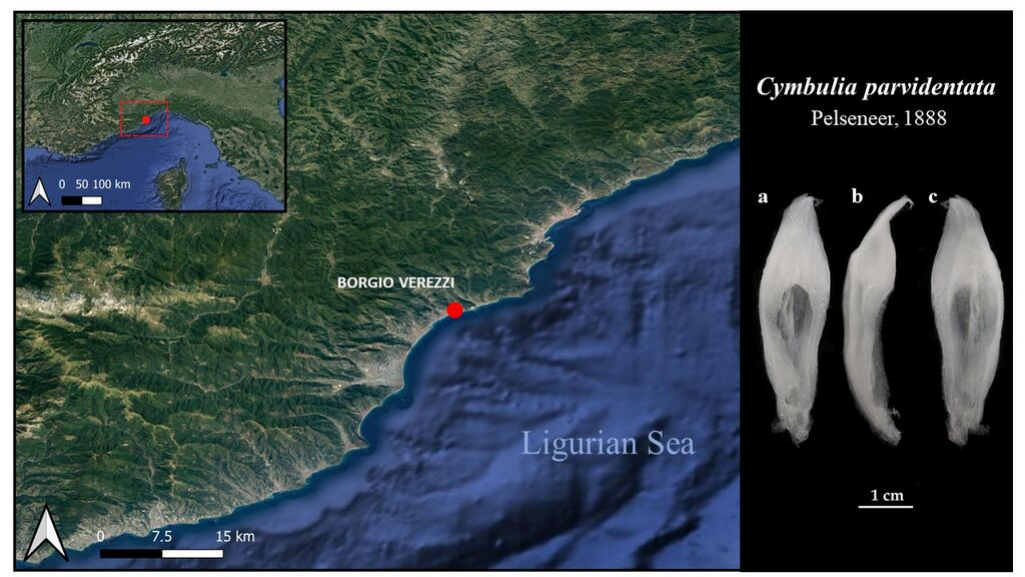
Study area with sampling site for beached specimens. Specimen of *Cymbuliaparvidentata* - frontal view (**a**), lateral view (**b**) and ventral view (**c**).

On 27/2/2022, seven stranded pseudoconchs belonging to the *Cymbulia* genus were collected on the beach of Borgio-Verezzi (44.16° N, 8.304633° W, Savona, Italy) between 9:00 and 10:30 AM (Fig. 1). Collected pseudoconchs were preserved in ethanol (96% Et-OH) for analyses. Species were characterised morphologically, based on available literature (Occhipinti Ambrogi 1989). Morphometric measures were also taken (cm ± SD): length, width, height and wet weight (WW, g ± SD). Besides size differences, clear morphological characteristics allow the identification of the two species. In detail: *C.peronii* presents a shell with a rather broad cavity and does not exhibit any narrowing in the middle of its length, spines bounding the aperture being present (larger on the right than on the left) (Pelseneer 1965, Occhipinti Ambrogi 1989). In *C.parvidentata*, the shell shows a very narrow cavity with small uniform spines on the surface, even on the borders of the aperture. A clear constriction in the median part of the pseudoconcha length characterises this species (Pelseneer 1965, Mondello and Rindone 1994). Wind direction and intensity data of the sampling day were obtained from the Liguria Region web page (https://www.regione.liguria.it/servizi/item/14279-banca-dati-meteoclimatica.html). Distributional data of the identified species were obtained from global open-access data repository (OBIS www.obis.org and GBIF www.gbif.org).

## Results

**Figure 2.**
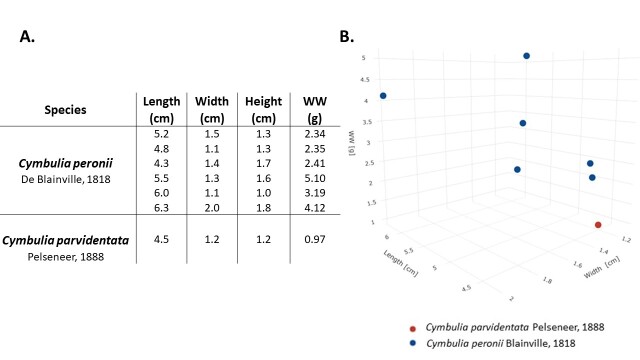
Morphometric measurements of analysed samples. **A** Biometric data for the two species of Cymbuliidae; **B** Three-dimensional scatterplot with biometric data relating to *C.peronii* (in blue) and *C.parvidentata* (in red).

Of the seven pseudoconchs analysed in this study, six belonged to *Cymbuliaperonii* and one to *Cymbuliaparvidentata* (Fig. 1). Morphometric measurements (Fig. 2) show that *C.peronii* has a length of 5.35 ± 0.75 cm, width is 1.4 ± 0.31 cm, height of 1.45 ± 0.29 cm, wet weight (WW) of 2.5 ± 1.35g. *C.parvidentata* has a length of 4.5 cm; width of 1.2 cm; height is 1.2 cm and a WW of 0.97 g. The two species can be distinguished from a morphological point of view (Fig. 2), *C.peronii* being larger, voluminous and consequently with a higher biomass than the other species. During the day of 27/2/2022, winds blew with a predominant direction between W/NW and E/NE. The most frequent wind was the NW one with an average speed of 3.325 ± 1.11 m/s^2^ (max = 5.2 m/s^2^, min = 0.8 m/s^2^).

## Discussion

Pteropods play a very important role in the biogeochemical and trophodynamic cycles (Manno et al. 2019) where they represent the link between lower trophic levels and secondary consumers (Koppelmann et al. 2013). They also represent efficient markers of different water masses (Chen and Bé 1964). The stranding phenomenon of these pteropods along Italian coasts is already historically known and may be related to biological, oceanogeographic and meteorological processes (Battaglia et al. 2020, Bergamasco et al. 2022). Generally, the environmental determinants that influence the beaching of zooplankton species are wind direction and intensity, surface circulation of sea currents and temperature (Boero 1994). The stranding of specimens belonging to the Cymbuliiade family, locally known as "Scarpetta di Venere" (i.e. “Venus’ shoes”), is a known, but rarely documented phenomenon (Mondello and Rindone 1994). While the records of *C.peronii* strands in the Ligurian Sea are not rare and sometimes specimens are stranded in high numbers (e.g. Occhipinti Ambrogi (1989)), *C.parvidentata* is reported for the first time in this area. This species, in fact, is known to sporadically occur only in the southern Tyrrhenian and Ionian Seas (Cattaneo-Vietti and Giovine 2008) where it is considered a marker of AW (Furnestin 1979) masses that enter the Strait of Gibraltar. Our finding suggests that these AW ingressions may extend to reach the Ligurian Sea, one of the colder Mediterranean areas, which is now facing a progressive and apparently unstoppable warming. This hypothesis is corroborated by the previous findings of other Atlantic species, such as *D.fascicularis* (Betti et al. 2017). In the next years, it will be important to systematically record strandings of *C.parvidentata* and of other species of Atlantic origin in the Ligurian Sea. Due to the length of the Ligurian coasts, this “sentinel system” could only be realised with the help of citizens. It is, thus, highly desirable that *ad hoc* citizen-science campaigns could be designed to monitor these on-going changes.

## Conclusion

We report the presence and biometric characteristics of two pseudoconchs species of Cymbuliidae, *Cymbuliaperonii* and *Cymbuliaparvidentata*, found on the beach of Borgio-Verezzi (Savona, Italy). Our finding reports a first record in the Ligurian Sea and represents a baseline for future investigation regarding the pteropods community in the area. In this work, we highlight the valuable resource that ad hoc citizen-science projects will represent in the context of seasonal strandings monitoring.
